# Antioxidant Activity-guided Phytochemical Investigation of *Artemisia aucheri* Boiss.: Isolation of Ethyl Caffeate and a Spinacetin Glycoside

**DOI:** 10.22037/ijpr.2019.15496.13140

**Published:** 2021

**Authors:** Parvin Jahanbani, Sajjad Nasseri, Mahdi Mojarrab

**Affiliations:** a *Students Research Committee, Kermanshah University of Medical Science, Kermanshah, Iran. *; b *Department of Pharmacognosy and Pharmaceutical Biotechnology, School of Pharmacy, Kermanshah University of Medical Sciences, Kermanshah, Iran. *; c *Pharmaceutical Sciences Research Center, Health Institute, Kermanshah University of Medical Sciences, Kermanshah, Iran.*

**Keywords:** *Artemisia aucheri*, Total phenolic content, Free radical scavenging activity, Ferrous ion chelating activity, Vacuum liquid chromatography, Ethyl caffeate, Spinacetin glycoside

## Abstract

Antioxidant activity of five different extracts (petroleum ether, dichloromethane, ethyl acetate, ethanol and ethanol-water) of *Artemisia aucheri* aerial parts was investigated by three various methods: ferrous ion chelating (FIC) assay, 2,2-diphenyl-1-picrylhydrazyl (DPPH) radical scavenging method and β-carotene bleaching (BCB) test. Total phenolic contents (TPC) were measured by Folin–Ciocalteu method. The hydroethanolic extract exhibited the stronger inhibitory activity in BCB and FIC assays than the other extracts. Among the extracts analyzed, the ethyl acetate and ethanolic extracts exhibited the highest TPC and DPPH radical scavenging activity, respectively. Reversed phase vacuum liquid chromatography of ethanolic extract (with the highest extraction yield) produced five fractions (A to E) which were subjected to all antecedent experiments. The same sample (Fraction C) showed the highest TPC and DPPH radical scavenging activity while there were no statistically significant correlations between TPC and EC_50_ values of various antioxidant assays. Ethyl caffeate and a spinacetin glycoside were isolated from the most active fraction and their structures were established using spectroscopic analysis including NMR and MS.

## Introduction

Within the important plant family, Compositae (Asteraceae), *Artemisia *is a genus of small herbs and shrubs and comprises over 500 species, which are generally found in Europe, Asia and North America (1).* Artemisia aucheri* *(A. aucheri) *Boiss called ‘’Dermaneye koohi’’ in Persian language and grows rather abundantly in Iran (2). Some oxygenated monoterpenoids like camphor (3), verbenone (4), linalool (5, 6), 1,8- cineol (7), α- thujone (8), and borneol (9) were identified as major components in the volatile oil. Normal phase preparative TLC resulted in the isolation of six highly oxygenated monoterpenoids (10). Research on the volatile oil and crude extract has demonstrated *in-vitro *acaricidal (11), antimicrobial (12), and antifungal (5, 13 and 14) effects. *A. aucheri *extracts have also shown *in-vitro *and *in-vivo *anti-leishmanial activity (15-17). Dichloromethane extract of the species has been effective in a cell-free antimalarial assay (18). In contrast, the ethanolic extract was active in an* in-vivo *method (19). Cytotoxic (20, 21), hepatoprotective (22), skin wound healing (23), hypocholesterolemic and antiatherosclerotic effects (24, 25) have been reported from various extracts of *A. aucheri. *Recently, cytotoxic sesquiterpenoids have been isolated from the petroleum ether extract of the species (26).

Based on the various observed antioxidant effect of essential oil, methanolic and aqueous extracts in previous reports, assessment of antioxidant activity of several extracts and fractions by different methods and isolation of major compounds from the most active fraction was carried out in the current study (27-29).

## Experimental

*Reagents and chemicals*

β- Carotene and 1,1-diphenyl-2-picryl-hydrazyl (DPPH) were purchased from Sigma- Aldrich. Linoleic acid, gallic acid, ferrous chloride, sodium carbonate, dimethyl sulfoxide (DMSO), chloroform, Tween^®^40, Folin-Ciocalteu’s phenol reagent, ethylenediaminetetraacetic acid (EDTA), butylatedhydroxytoluene (BHT), LiChroprep^®^RP-18 (15-25 μm) were purchased from Merck, ascorbic acid from VWR, ferrozine iron reagent from Acros Organics and all the solvents used for extraction and purification procedures were of gradient grade and purchased from Scharlau (Spain) and Caledon (Canada).

*General experimental procedures*

The chromatographic system for semi-preparative HPLC consisted of a binary pump YL 9111S, a PDA detector YL9160 and a VertiSep UPS C18 (250 × 30 mm i. d., 10 μm) column. AnAscentis^®^ (250 ×10 mm i. d., 5 μm) column was replaced for final purification. NMR spectra were recorded on a Bruker AVANCE III 500 MHz spectrometer in dimethyl sulfoxide-d_6_as, the solvent and the residual solvent signal used as an internal standard. ESIMS data were obtained on an Esquire 3000 plus ion trap mass spectrometer (Bruker).

*Plant material*

Aerial parts of the plant were collected from ChaharBagh region, Gorgan (Golestan province, Iran) in September 2011. The sample was identified by Mr. S. A. Hosseini, Agricultural and Natural Resources Research Center of Golestan Province, Gorgan, Iran. A voucher specimen (number 2383) is deposited in the herbarium.

*Extraction and isolation*

Air-dried and ground aerial parts (200 g) of* A. aucheri *were extracted with petroleum ether (40-60), dichloromethane, ethyl acetate, ethanol and ethanol-water (1:1 v/v) respectively (Sequential maceration with ca. 3 × 2.0 L of each solvent). The extracts were filtrated with filter paper and dried using a rotary evaporator at a reduced pressure at a temperature below 45 ◦C to yield 2.22, 8.92, 0.92, 18.18 and 14.54 g of the corresponding extracts, respectively. Six grams of the selected sample in DPPH assay (ethanolic extract) was subjected to a reversed-phase vacuum liquid chromatography (VLC) using a step gradient of MeOH-H_2_O (1:9, 2:8, 4:6, 6:4, 8:2, 10:0, 600 mL each) to give five fractions (A, B, C, D and E) respectively (Table 1). Fraction C (603 mg) which rich in phenolic components was re-fractionated by semi-preparative HPLC (mobile phase: 0–30 min, MeOH from 40 to 70% in H2O; 30 –35 min, 70% MeOH in H2O; 35 –37 min MeOH from 70 to 100% in H2O; 37–47 min 100% MeOH, flow rate 10 mL/min) to yield 13 subfractions.Further purification of the subfraction 12 (47.2 mg, *t*R = 29.9 min) by semi-preparative HPLC (mobile phase: 0–30 min, MeOH from 25 to 55% in H2O; 30–35 min, 55% MeOH in H2O; 35–36 min MeOH from 55 to 100% in H2O; 36–40 min 100% MeOH, flow rate 3 ml/min) yielded compounds **1 **(4.2 mg, *t*R = 28.7 min). Further purification of the subfraction13 (3.6 mg, *t*R = 35.9 min) by semi-preparative HPLC (mobile phase: 0–30 min, MeOH from 30 to 60% in H2O; 30–35 min, 60% MeOH in H2O; 35–36 min MeOH from 60 to 100% in H2O; 36–40 min 100% MeOH, flow rate 3 mL/min) yielded compounds **2 **(1.8 mg, *t*R = 29.5 min).

*Antioxidant assays and Measurement of total phenolic contents*

*Total phenolic contents*

The total phenolic content (TPC) was measured by the Folin–Ciocalteu method with some modification. Different concentrations of samples in water (0.5 mL) were mixed with 2.5 mL of Folin- Ciocalteu reagent (0.2 N) (30, 31). Two milliliters of Na_2_CO_3 _solution (75 g/L) was added after 5 min. After 2 h standing in the dark, the optical density was measured at 760 nm against a blank. The total phenolic contents were calculated based on gallic acid calibration curves and expressed as milligrams of gallic acid equivalents (GAE), per gram of dried samples.

*DPPH radical scavenging activity*

The assay was performed according to the method of Hatano *et al.* with slight modifications (32). Briefly, test samples were dissolved in methanol at different concentrations. Equal volumes of 0.2 mM solution of DPPH in methanol were added to each of the test tubes. The mixture was shaken vigorously and maintained in the dark for 30 min. Then, the absorbance was read at 517 nm against a blank. Butylated hydroxytoluene (BHT) and ascorbic acid were used as standard references. The scavenging activity was calculated using the formula:

I% = (A_C–_A)/A_C_ × 100 

Equation 1.

Where A_c _= absorbance of the control and A = absorbance of a tested sample in 30 min.

*Metal chelating activity*

The chelating activity of extracts and fractions for ferrous ions Fe^2+ ^was measured adopting the ferrous iron– ferrozine complex method with some modification (33). Briefly, 25 µL of FeCl_2 _solution (2 Mm) was added to a mixture containing 2 mL of methanolic solution of the test sample and 1.5 mL of H_2_O. The reaction was started by adding 50 µL of ferrozine solution (5 mM) to each test tube after 30 sec. The mixtures were shaken well and incubated for 10 min at room temperature. The absorbance of the solution was then read at 562 nm. EDTA and quercetin were used as positive controls. The ability of the samples to chelate ferrous ion was calculated using the equation mentioned above for DPPH radical scavenging activity.

*Inhibition of* *β*-*carotene bleaching*

The antioxidant potential of the extracts and fractions were determined by a slightly modified version of the β-carotene bleaching method (34). Linoleic acid (33 µL) was added to 225 mg of Tween 40 and 750 µL of β-carotene solution (0.500 mg/mL). The solvent was completely removed using a rotary evaporator. After adding 75 mL of oxygenated distilled water, the mixture was emulsified for 15 min in a sonicator to give emulsion A. Aliquots of 3.5 mL of this emulsion were transferred into a series of stopper test tubes containing 1 mL of samples dissolved in water or DMSO in different concentrations. Optical density (OD) at 470 nm was recorded for all samples immediately (t = 0) and at the end of the assay time (t = 120). An emulsion composed of 50 mL of oxygenated water, 22 µL of linoleic acid and 150 mg of Tween 40 was also prepared to use as the blank to zero the spectrophotometer. The percentage inhibition was calculated according to the following formula: 

I% = (A_A(120) - _A_C(120)_ )/(A_C(0) _- A_C(120) _) 100 ×

Equation 2.

Where A_A(120) _is the absorbance of the sample at t = 120 min, A_C(120)_ is the absorbance of the control at t = 120 min, and A_C(0) _ is the absorbance of the control at t = 0 min.

*Statistical analysis*

All the experiments were performed in triplicate. The data were reported as mean ± standard deviation (SD) (n = 3) and evaluated by non parametric Friedman test. The difference was considered to be statistically significant if *P* < 0.05. Pearson’s correlation coefficients (r) between total phenolic contents of the samples and calculated EC_50_ values were determined in each antioxidant assay.

## Results

*Extraction and isolation*

**Figure 1:**
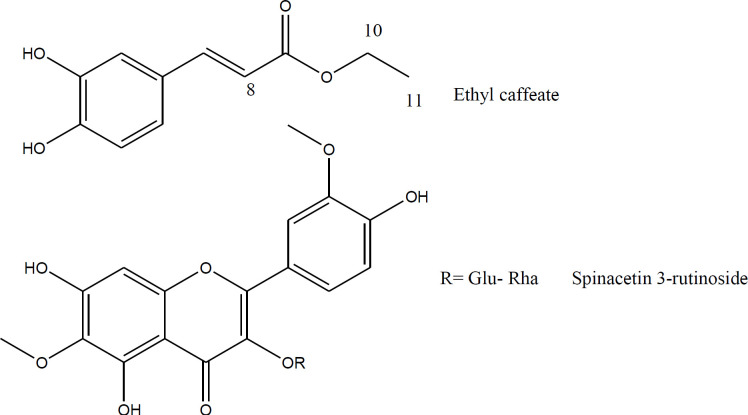
Chemical structures of isolated compounds

Fractionation of the ethanolic extract by a combination of VLC and semi-preparative HPLC on RP-18 afforded compounds 1 and 2 (Figure 1). The chemical structures of compound 1 and aglycone of the compound 2 were elucidated unequivocally through ESIMS and NMR and also all spectroscopic data were in agreement with respective published data (35-37). The structure of glycoside moiety in compound 2 was tentatively assigned due to the lack of experimental data on acid hydrolysis and successive sugar identification by comparison with authentic samples.

Compound 1 (Ethyl *trans- *caffeate): ESI-MS (m/z): 207.1 [M-H]^-^, 415.1 [2M-H]^-^. ^1^H NMR (500 MHz, DMSO-d_6_) δ (ppm): 1.25 (3H, t, *J *= 7.05 Hz, H-11), 4.16 (2H, q, *J *= 7.05 Hz, H-10), 6.24 (1H, d, *J *= 16.00 Hz, H-8), 6.76 (1H, d, *J *= 7.70 Hz, H-5), 6.98 (1H, d, *J *= 7.70 Hz, H-6), 7.05 (1H, s, H-2); 7.47 (1H, d, *J *= 16.00 Hz, H-7),^13^C-NMR (data from HSQC and HMBC spectra, DMSO-d6) δ (ppm): 15.2 (C-11), 60.2 (C-10), 114.5 (C-8), 115.2 (C-2), 116.1 (C-5), 121.8 (C-6), 126.0 (C-1), 145.7 (C-7), 146.2 (C-3), 149.2 (C-4), 168.0 (C-9).

Compound 2 (Spinacetin 3-rutinoside): ESI-MS (m/z): 653.6 [M-H]^-^, 655.5 [M+H]^+^, 677.4 [M+Na]^+1^. H NMR (500 MHz, DMSO-d_6_) δ (ppm): 1.00 (3H, d, *J *= 6.2 Hz, H-6”’), 3.08 (2H, m, H-4” and H-4”’), 3.20–3.35 (7H, m, H-3”, H-2”, H-5”, H-5”’, H-3”’, H- 6”_b_, and H-2”’), 3.73 (1H, m, H-6”_a_), 3.77 (3H, s, 6-OMe), 3.85 (3H, s, 3’-OMe), 4.43 (1H, br s, H-1”’); 5.42 (1H, d, *J *= 7.1 Hz, H-1”), 6.51 (1H, s, H-8), 6.92 (1H, d, *J *= 8.4 Hz, H-5’), 7.52 (1H, dd, *J *= 8.4 , 1.4 Hz, H-6’), 7.84 (1H, d, *J *= 1.4 Hz, H-2’),^ 13^C-NMR (data from HSQC and HMBC spectra, DMSO-d6) δ (ppm): 18.1 (C-6”’), 56.4 (3’-OMe), 60.3 (6-OMe), 67.3 (C-6”), 68.7 (C-5”’), 70.7 (C-4”), 71.2 (C-2”’ and C-3”’), 72.4 (C-4”’), 74.3 (C-2”), 74.9 (C-5”), 76.7 (C-3”), 94.6 (C-8), 101.3 (C-1”’), 101.8 (C-1″), 113.7 (C-2’), 115.8 (C-5’), 121.4 (C-1′), 122.6 (C-6′), 132.0 (C-6), 133.2 (C-3), 147.1 (C-3’), 150.2 (C-4’), 156.6 (C-2), unobserved signals (C-4, C-5, C-7, C-9 and C-10).

*Total Phenolic Content*

The regression equation of the calibration curve of gallic acid (R^2^ = 0.997, *y *= 0.011*x* + 0.057) was used to calculate the content of phenolic compounds and expressed in GAE as milligrams per gram of each sample (mg GAE/g extract or fraction). TPC of the samples showed large variations, between 3.67 ± 2.52 (DCM extract) and 338.23 ± 4.22 (fraction C) mg GAE/g extract or fraction (Table 1).

*DPPH radical scavenging activity*

Except for fraction A and petroleum ether and dichloromethane extracts, all the samples showed moderate to good scavenging performance on DPPH assay. The highest activity was observed for fraction C, with the EC_50_ value of 18.75 ± 0.07 µg/mL, followed by the fractions B and D with the EC_50_ values of 27.71 ± 0.36 and 37.40 ± 0.11 µg/mL, respectively (Table 1).

*Metal chelating activity*

The only active sample in the FIC method was hydroethanolic extract with the EC_50_ value of 157.62 ± 0.82 µg/mL (Table 1).

*Inhibition of *β-*carotene bleaching*

Fraction D showed the best inhibitory performance, with an EC_50_ value of 7.90 ± 0.81 μg/mL while fraction A (EC_50 _= 127.61 ± 5.98 μg/mL) exhibited the lowest (Table 1).

*Statistical analysis*

Pearson’s correlation coefficients between the TPC and calculated EC_50 _values for DPPH, FIC and BCB assays took the values of -0.574, 0.052 and -0.106, respectively. The lowest correlation was observed between the TPC of the samples and their capacity to chelate ferrous ions. There were no significant correlations between TPC and DPPH radical scavenging activities of the samples and their ability to inhibit the bleaching of *β-*carotene. The Friedman test results demonstrated that neither the DPPH assay nor the BCB test had significantly different results in screening the samples for their antioxidant ability.

## Discussion

To the best of our knowledge, this is the first report on the presence of ethyl caffeate and spinacetin 3- rutinoside in *A. aucheri.* The structures of isolated compounds were elucidated by ESIMS, ^1^H- and 2D-NMR and compared with spectroscopic data reported in the literature (35-37). The first compound showed the pseudo-molecular-ion peak at m/z 207.1 ([M-H]^-^), representing the molecular formula C_11_H_12_O_4_. The ^1^H NMR spectrum of the compound revealed the presence of a 3, 4- dihydroxycinnamoyl moiety with *trans*-geometry of the double bond (*J* = 16.0 Hz). The signals of the aromatic H-atoms (H-2, H-5, and H-6) in compound 1 were observed between 6.76 and 7.05 ppm. The HMBC correlations between the H-atoms of caffeic acid (H-2, H-5, and H-6) and C-3 or C-4, as well as the same correlation between H-8 and C-9 were used to determine the chemical shift of the carbon atoms. The ESIMS pseudo-molecular-ion peak of compounds 2 was observed at m/z 653.6 ([M-H]^-^), consistent with the molecular formula of C_29_H_34_O_17_. In the ^1^H-NMR spectrum of compound 2, the signals of the aromatic H-atoms (H-8, H-2’, H-5’, and H-6’) resonated between 6.51 and 7.84 ppm. In the HSQC spectrum, these protons showed direct correlations with four distinctive carbon chemical shifts at 94.6, 113.7, 115.8, and 122.6 ppm which suggested a 5, 6, 7, 3′, 4′ pentasubstituted flavonol skeleton. In the HMBC spectrum, the methoxy group signals at chemical shifts of 3.77 and 3.85 ppm correlated with the carbon atoms at the chemical shifts of 132.0 and 147.1 ppm, respectively indicating the presence of two methoxy groups at C-6 and C-3’. Thus, the aglycone part is quercetagetin 3’,6-dimethyl ether (Spinacetin).

In the ^1^H NMR spectrum of compound 2, signals of two distinctive anomeric protons were observed, which suggested that compound 2 is a flavonol disaccharide. In the HSQC spectrum, the sugar carbon signals were in good agreement with the reported values for (-α-L-rhamnopyranosyl-(1→6)-β-D-glucopyranoside) (38, 39). The observed correlation in HMBC spectrum between H-1’ and C-6” supported the suggested structure. The complete NMR spectroscopic data for the spinacetin 3-rutinoside was not found in the literature. Comparison of recorded NMR values to those reported for spinacetin 3-O- robinobioside, as one of the most possible alternatives to the proposed structure, showed notable differences in proton and carbon chemical shifts of H-2” to H-5” and C-2” to C-6”, respectively (36). However, the small amounts of compound 2 isolated precluded the performing acid hydrolysis and sugar identification by comparison with authentic samples. These facts, along with comparing the rest of spectroscopic data with those reported in the literature, allowed the structure elucidation of compound 1 as ethyl-*trans*- caffeate, and tentative identification of compound 2 as spinacetin 3-rutinoside, respectively (35-37).

The compounds have been previously isolated from other genus *Artemisia* species such as *Artemisia minor* (*A. minor),* *Artemisia incisa (A. incise),* and *Artemisia absinthium (A. absinthium) *(40-42)*. Artemisia copa (A. copa) *has been another species in the genus which is known as a source of the aglycone of spinacetin (43). To the best of our knowledge, there is only one previous report of isolation and identification of the compound 2 from Iranian flora (44). Spinacetin as one of the contributors to antioxidant capacity of spinach leaves exhibited considerable antioxidant activity in the DPPH assay (45,46). Spinacetine gentiobioside exerted moderate ABTS radical-scavenging activity and comparable with the activity of BHT (47). Spinacetin-3-O-robinobioside has been reported as one of the constituents of a fraction with the most pronounced DPPH radical scavenging activity from *Oxybaphus* *nyctagineus *(48).

The presence of ethyl caffeate in the genus *Artemisia* has led to *in -vitro* cytotoxicity against the HepG2 cancer cell line (40). Ethyl caffeate has a valuable effect on scavenging of superoxide anion, nitric oxide and DPPH radical and it also significantly inhibits hydrogen peroxide-induced neuronal PC12 cell death at 5 and 25 μM (49, 50). Ethyl caffeate has been previously isolated from *Elsholtzia densa, Ipomoea batatas*, and *Ilex latifolia* due to antioxidant activity- guided phytochemical studies (51-53). In the current study, DPPH radical scavenging activities of the samples displayed a better correlation to their total phenolic contents, as clarified by the Pearson’s correlation coefficients. The Friedman test results were consistent with the last report for* Artemisia biennis* *(A. biennis)* (54), which suggest DPPH and BCB assays are not significantly different in the selection of active samples. The potent scavenging activity of ethanolic extract of *A. aucheri *on DPPH radical and its high-yield preparation was in contrast with the results of similar studies on *A. biennis *and* Artemisia ciniformis* *(A. ciniformis)*. TPC of ethanolic extract of *A. aucheri* was higher than those of similar extracts of *A. biennis *and *A. ciniformis, *as well (54, 55). These findings might partly be related to the presence of different types of phytochemicals in *A. aucheri.*

**Table 1:** Antioxidant performance and total phenolic contents of the extracts/fractions from A. aucheri

**Sample**	**Extraction/fractionation yield (g)**		**EC**_50 _ **(µg/mL)**				**TPC** **(mg GAE/g)**
			**DPPH assay**	**FIC assay**	**BCB assay**		
PE	2.22		1026.01 ± 89.55	-	67.24 ± 0.43		13.33 ± 0.58
DCM	8.92		348.02 ± 14.06	-	28.61 ±0.69		3.67 ± 2.52
EA	0.92		44.07 ± 0.78	-	53.73 ± 1.36		156.33 ± 2.02
EtOH	18.18		41.87 ± 0.57	-	19.15 ± 1.36		142.60 ± 2.50
EtOH/Wt	14.54		68.08 ± 0.80	157.62 ± 0.82	12.13 ± 0.16		103.27 ± 1.33
Fr. A	2.97		124.87 ± 0.33	-	127.61 ± 5.98		45.49 ± 0.83
Fr. B	0.49		27.71 ± 0.36	-	47.11 ± 3.93		211.28 ± 0.62
Fr.C	1.16		18.75 ± 0.07	-	55.49 ± 7.47		338.23 ± 4.22
Fr. D	0.75		37.40 ± 0.11	-	7.90 ± 0.81		103.30 ± 0.96
Fr. E	0.28		59.29 ± 0.18	-	8.92 ±0.50		94.80 ± 0.36
BHT	---		4.96 ± 0.66	---	0.469 ± 0.22		---
Vit C	---		4.74 ± 0.19	---	---		---
EDTA	---		---	18.94 ± 2.88	---		---
Quercetin	---		---	88.35 ± 4.09	---		---

## Conclusion

DPPH radical scavenging activities of *A. aucheri *ethanolic extract and some of its derived fractions in comparison with other samples could be ascribed to their higher content of phenolic compounds like caffeic acid derivatives and glycosylated flavonols which were isolated in this study. 
